# Dispersion fields reveal the compositional structure of South American vertebrate assemblages

**DOI:** 10.1038/s41467-019-14267-y

**Published:** 2020-01-24

**Authors:** Michael K. Borregaard, Gary R. Graves, Carsten Rahbek

**Affiliations:** 1 Center for Macroecology, Evolution and Climate, GLOBE Institute, University of Copenhagen, Universitetsparken 15, 2100 Copenhagen, Denmark; 2 Department of Vertebrate Zoology, National Museum of Natural History, Smithsonian Institution, Washington, DC 20013 USA; 3 Imperial College London, Silwood Park, Buckhurst, Road, Ascot, Berkshire, SL5 7PY UK; 4 Danish Institute for Advanced Study, University of Southern Denmark, Odense M, 5230 Denmark

**Keywords:** Biogeography, Macroecology

## Abstract

The causes of continental patterns in species richness continue to spur heated discussion. Hypotheses based on ambient energy have dominated the debate, but are increasingly being challenged by hypotheses that model richness as the overlap of species ranges, ultimately controlled by continental range dynamics of individual species. At the heart of this controversy lies the question of whether species richness of individual grid cells is controlled by local factors, or reflects larger-scale spatial patterns in the turnover of species’ ranges. Here, we develop a new approach based on assemblage dispersion fields, formed by overlaying the geographic ranges of all species co-occurring in a grid cell. We created dispersion fields for all tetrapods of South America, and characterized the orientation and shape of dispersion fields as a vector field. The resulting maps demonstrate the existence of macro-structures in the turnover of biotic similarity at continental scale that are congruent among vertebrate classes. These structures underline the importance of continental-scale processes for species richness in individual assemblages.

## Abstract (web summary)

Ecologists continue to debate whether local species assemblages result from habitat filtering or from turnover among the regional species pool. Here the authors develop a “dispersion field” method to mapping species range overlaps, showing that regional turnover processes are key to local assembly.

## Introduction

**Fig. 1:**
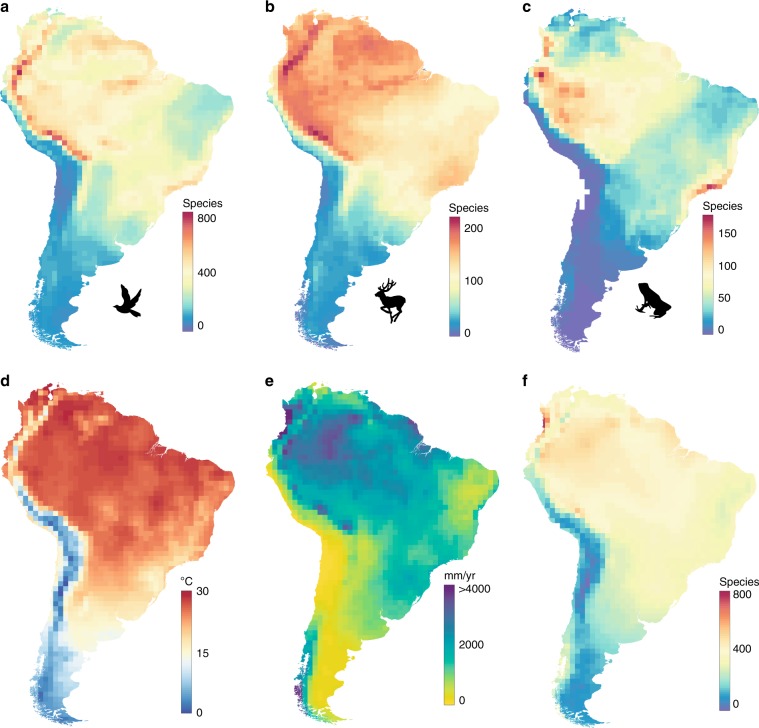
Species richness and environmental variables in South America mapped in 1° × 1° grid cells. **a** Species richness of birds (*n* = 2869), **b** mammals (*n* = 1146), and **c** amphibians (*n* = 2265). **d** Mean annual temperature (°C). **e** Annual precipitation (mm). **f** Predicted richness of birds based on a linear model of temperature and precipitation (*R*^2^ = 0.63).

The cause of continental patterns of species richness is one of the most debated questions in ecology^1–3^ and was for years considered the keystone question of macroecology. Though species richness of lowland regions generally correlates well with contemporary climate^4^, with mountainous regions correlating less well (refs. ^5,6^, Fig. 1), the mechanistic processes responsible for this correlation remain unresolved^7,8^. Hypotheses based on a direct local effect of ambient energy on species richness have dominated the debate, countered by theories focusing on species’ niches and range dynamics, positing that species richness is an emergent property of species range overlap^9–13^ that occurs at scales much greater than the grid cells used in most analyses. An example of the latter is the idea of tropical niche conservatism, which posits that climate–richness correlations arise because many species are evolutionarily adapted to warmer and more productive environments and thus have ranges extending into, or confined to, these areas. Since these hypotheses make similar predictions for spatial variation in species richness, a resolution to this controversy has not been found, and in recent years relatively little progress has been made on the processes underlying patterns of large-scale species richness.

Though mechanisms based on (1) energy limiting rates of species origination^14^, (2) energy limiting local co-occurrence^15^, or (3) richness arising as an emergent property of species’ niche overlap^16^ will all lead to the observed correlation between climate and richness, they represent three different underlying pathways for the generation of continental species richness patterns. We argue here that these differences should be reflected in the continental pattern of species’ turnover among grid cells, making it possible to distinguish between them.

Energetic limitation of speciation rates has generally been tested by correlating species richness with local energy levels^4^ or by phylogenetically estimating the past diversification rates of all species whose current ranges overlap a given grid cell. Both of these approaches will lead to biased results in the presence of spatio-temporal range dynamics. Because speciation in terrestrial vertebrates is usually allopatric^17^, newly geminate species will generally not co-occur, and thus the species richness of grid cells only increases where the species come into secondary contact. This may accumulate species in the area of origination, but if range expansion is not spatially random species richness will accumulate in the area that ranges expand into, which will greatly weaken the expected spatial link between local speciation rates and species richness. An empirical example of this is the high salamander diversity of Amazonia, which is mainly generated by species diversification in the Andes^18^. Thus speciation rate variation will only lead to strong energy–richness correlations when range expansion is relatively spatially symmetric around the area of origination.

Energetic limitation of species co-occurrence at large spatial scales^19^ is an alternative local species–energy mechanism. This hypothesis is based on the theoretical premise that range expansion occurs more easily in areas with high energy and many resources and that local extirpation and range contraction is more likely in areas with less available energy. This is predicted to lead to range dynamics that are similar across different species, with high-energy areas as foci for all species range expansions.

Hypotheses based on range overlap, such as Tropical Niche Conservatism, in contrast, explicitly posit that range expansion history is determined by the spatial configuration of the landscape and underlies species richness patterns. Under the mechanism of niche conservatism, species with similar adaptations are likely to co-occur deterministically across sites, leading to large-scale emergent patterns of range overlap and geographically structured assemblages whose spatial distribution follow the asymmetric configuration of continental structures, such as the turnover of ecoregions characterized by different biomes^20–22^. These processes are predicted to leave a distinct biogeographical signature in the continental pattern of species distributions. Because these patterns result from a deterministic interaction with the physical environment, the pattern of disequilibrium should be predictable and consistent among taxa with shared habitat affinities.

The existence of geographically structured assemblages with a clear signature of biomes would thus support a key prediction of niche conservatism. Note that the well-established existence of biogeographic regions in species distributions^23^ do not in themselves invalidate species–energy theory; however, as outlined above, the energy-based mechanisms will predictably affect spatial patterns of biotic similarity, giving us a previously unexplored opportunity to reassess the mechanistic basis of climate–richness correlations.

Here we develop a new approach to reveal and visualize geographic structuring of assemblages. The approach is based on assemblage dispersion fields, which are formed by overlaying the geographic ranges of all species co-occurring in a grid cell. We created dispersion fields for all tetrapods of South America and visualized their geographic structures by expressing their orientation and shape of dispersion fields as a vector field. These symmetry diagrams support the existence of macrostructures in the turnover of biotic similarity at a continental scale. The structures are congruent among vertebrate classes, lending support to the predictions of niche conservatism as a control of continental diversity patterns.

## Results and discussion

### Assemblage dispersion fields (ADFs)

**Fig. 2:**
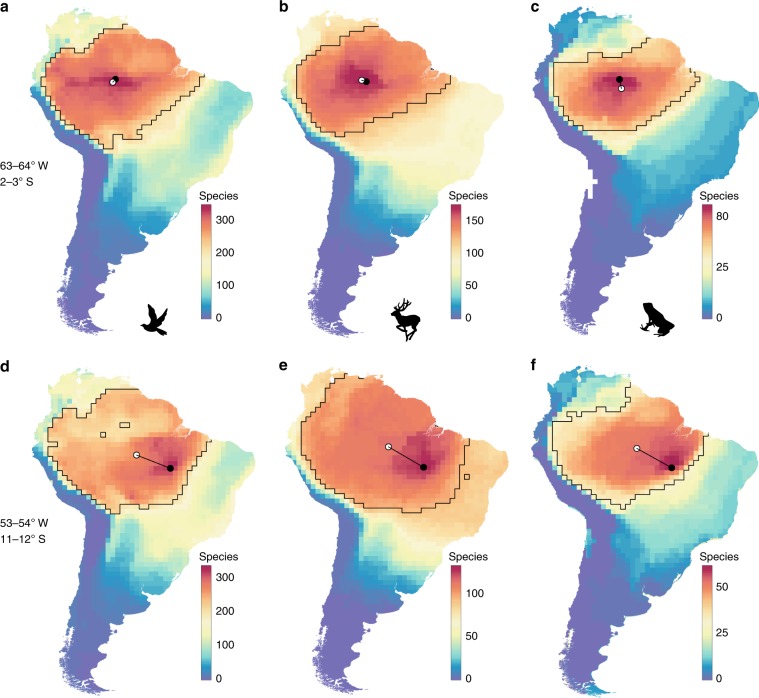
Asymmetry vectors and assemblage dispersion fields (ADFs) for selected 1° × 1° grid cells in Amazonia. The focal cell is marked by a black point. Colors indicate the number of species shared with the focal cell. The central region (see “Methods”) of the dispersion field is outlined by a black line. The asymmetry vector extends from the center of the focal cell to the center of gravity (white point) of the central region. ADFs of focal cells are relatively symmetrical near the center of the Amazonian ecoregion (**a**–**c**) and become increasingly asymmetrical near its periphery (**d**–**f**), for birds (**a**, **d**), mammals (**b**, **e**), and amphibians (**c**, **f**).

We investigated the geographical pattern of compositional similarity for the mammals, amphibians, and freshwater and land birds in 1689 cells (1° × 1° latitude–longitude blocks) in South America. To quantify patterns of compositional similarity, we used the approach of ADFs, which are created by overlapping the geographic range maps of all species that occur in a specified grid cell^24–27,28^. The resulting contour map will peak at the focal cell and decline in all directions from this peak (Fig. 2, Supplementary Figs. 1 and 2). The contour slope along any vector illustrates the decay of compositional similarity with distance from the focal cell, revealing the biogeographical affiliation of the species assemblage of the focal cell. We generated ADFs for all 1° × 1° grid cells (*n* = 1689) of the South American continent for all species of birds (2869 species), mammals (1146 species), and amphibians (2265 species).

Continental mapping of ADFs for individual grid cells reveals a clear geographical structure in the species composition of local assemblages^24^ in all three vertebrate classes (Fig. 2). The size and shape of ADFs exhibited marked geographic variation, reflecting patterns of faunal turnover and the spatial configuration of major biomes. For example, the composition of assemblages of species in 1° × 1° grid cells occurring at opposite ends of the vast Amazonian ecoregion (~5 million km^2^) are more similar to one another than they are to geographically proximate assemblages in adjacent ecoregions that exhibit similar energy levels. ADFs sampled from the center of the Amazonian ecoregion are relatively symmetrical (Fig. 2a–c), whereas those sampled near the periphery of the ecoregion are asymmetrical (Fig. 2d–f). We quantified ADF symmetry by projecting a vector from the geographic center of each focal cell to the respective center of gravity for each ADF (Fig. 2; see “Methods”). The vector toward the center of gravity is a simple representation of the orientation and degree of asymmetry of the ADF. The direction of the vector indicates the major axis of asymmetry of the ADF, whereas vector length provides a direct measure of the degree of ADF asymmetry. In this context, asymmetry refers to a deviation from radial symmetry of individual dispersion fields.

### Symmetry diagrams

**Fig. 3:**
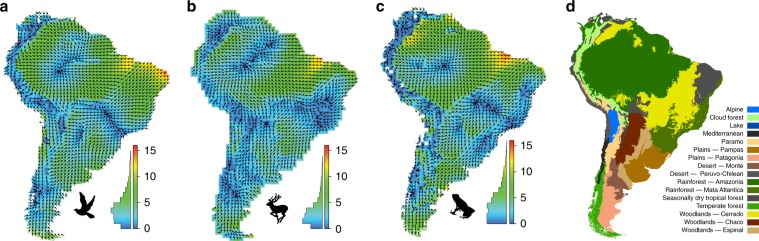
ADF symmetry diagrams for South American birds, mammals, and amphibians and major vegetation biomes. Arrows indicate the major axis of asymmetry of the ADFs for individual cells (*n* = 1689). The degree of asymmetry is indicated by a color scale (green through red colors represent more asymmetrical ADFs). This figure summarizes the vector arrows shown in Fig. 2 for all grid cells on the continent. Histograms show the distribution of vector lengths. The panels show **a** birds, **b** mammals, **c** amphibians, and **d** vegetation ecoregions. ADF symmetry diagrams using different cutoff values for the central ADF region are shown in Supplementary Figs. 3 and 5.). The map in **d** was provided by Tiina Särkinen, originally based on the map of the World’s ecoregions developed by the WWF (ref. ^48^; under CC BY 4.0 license: https://creativecommons.org/licenses/by/4.0/).

**Fig. 4:**
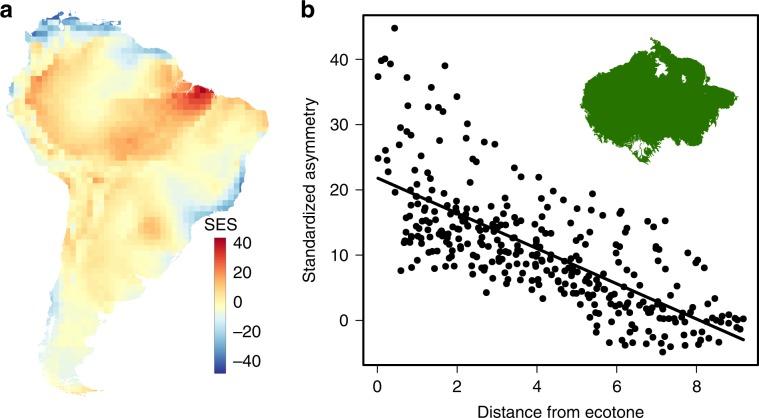
Null model divergence of ADF vector symmetry. **a** The deviation of the ADF symmetry vectors of birds from the null model (Supplementary Fig. 7), calculated as the standardized effect size (SES) deviation of vector lengths after 1000 repetitions of the null model. **b** The relationship between the deviation from the null model and the distance from the ecoregion boundary for the subset of grid cells (*n* = 342) that are completely encompassed within Amazonia (Supplementary Fig. 9; *R*^2^ = 0.52). The least square regression explains 52% of the variation.

We then mapped ADF symmetry diagrams for the continental lattice of cells using a color scale to illustrate the degree of ADF asymmetry and arrows to indicate the direction of the symmetry vectors (Fig. 3). This continental lattice of ADFs revealed a hidden biogeographical structure in the species composition of local assemblages that is not readily discerned from patterns of species richness (Fig. 3). For all three vertebrate groups, ADF symmetry diagrams revealed distinct geographical patterns in ADF symmetry and assemblage affinity (Fig. 3a–c). ADF vectors for the majority of adjacent grid cells tend to parallel one another or nearly so. However, ADFs near vegetation ecotones were highly asymmetrical, and the vectors of ADF symmetry diagrams straddling ecotones often point in orthogonal directions away from the ecotones and toward the centers of abutting ecoregions. In general, the degree of asymmetry increased with distance from these centers (Fig. 4). Groups of grid cells with similar ADFs correspond roughly to the configuration of major vegetation ecoregions (Fig. 3d), revealing, e.g., a clear separation of the faunas of Amazonian from the savannah-like biomes of the Cerrado and Caatinga, and an extremely rapid turnover of assemblages along the eastern versant of the Andes Mountains.

Note that, though the arrows on the diagrams might intuitively be understood as species movement, and indeed should to a degree reflect general patterns in range expansion, they do not prove movement and are not hypotheses about historical range dynamics for the faunas. The ADF symmetry diagrams are a representation of the current pattern of biotic similarity, whereas patterns of range expansion of individual species and clades can only be revealed by focused biogeographical analyses (and in the presence of fossils). Instead, the ADF symmetry diagrams reveal emergent structures in the current distribution of species that can be interpreted in the context of understanding the basis of the current pattern of species richness. The arrows reveal patterns at a larger scale than neighbor-based methods for compositional similarity, with the scale being determined by the species’ range sizes.

### Null model analysis

**Fig. 5:**
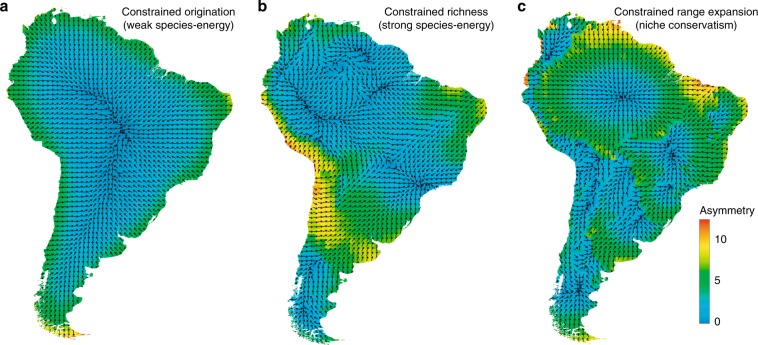
Predicted assemblage dispersion field (ADF) symmetry diagrams based on predictive models. The arrows reveal the pattern of biotic similarity. **a** The predicted ADF diagram from a model where species originate in grid cells according to local energy availability and then spread spatially randomly from there; **b** the predicted ADF diagram from a model where species richness is constrained to be identical to the empirical and ranges are constrained to be spatially cohesive; **c** the predicted ADF diagram from a model where each species originate in its original vegetation ecoregion and spreads spatially randomly with the constraint that ecoregion boundaries are crossed with low probability. Vegetation ecoregions are defined by the map shown in Fig. 3d.

We used a null model to correct for two properties of grid cells that are expected to affect ADF asymmetry in the absence of any ecological mechanism^29,30^: (1) The shape of the continent, which restricts the potential configuration of the ADF; and (2) the range–size frequency distribution of species in each grid cell, which will directly affect vector length, and thus asymmetry values. The null model assesses the expected ADF symmetry diagrams under random range placement (Fig. 5a) constrained by the empirical species richness (Fig. 5b) and ecoregion boundaries (Fig. 5c) (see “Methods”; Supplementary Fig. 7).

As a basis for interpreting the empirical ADF symmetry diagrams, we also developed simple predictive models under three constraints to operationalize the verbal models presented above: (1) species originate in grid cells with higher energy levels and undergo random range expansion (Fig. 5a); (2) species originate randomly and range expansion is limited by a maximum level of co-occurring species (Fig. 5b); and (3) species originate randomly within their native ecoregion and undergo range expansion limited by ecoregion boundaries (Fig. 5c). The emergent pattern in Fig. 5c is broadly similar to the empirical pattern. Note that this type of predictive model is necessarily an over-simplification and will to a certain extent reflect the choices made in implementing the algorithm; we present the results here mainly as a context for discussing the effect of various constraints on ADF symmetry diagrams.

## Conclusion

These results show that the species compositions of local assemblages are consistent with non-random range expansion limited by the geographical configuration of habitats, as suggested by niche theory. Though the patterns of ADF asymmetry are complex, they are consistent among three major vertebrate classes (Fig. 3). Thus range dynamics are not spatially random but instead result from deterministic processes that will weaken any spatial association between rates of speciation and species richness. Patterns of species richness appear instead to be created by an interaction between Grinnellian niches of species and geographical patterns of range overlap among species, which in turn depends on the configuration of major vegetation types. Furthermore, certain areas, such as the eastern Andes Mountains, exhibited very rapid faunal turnover, reflecting high turnover in species’ habitats. Grid cells from this area are consistently observed to support greater species richness than predicted purely from species–energy dynamics.

Our results support the idea that high species richness at the base of the Andes is due to high habitat heterogeneity and faunal turnover, rather than a simple response to elevated energy levels^31^. Our results show that, at the grain sizes of our analyses, the cohesive nature of the geographic ranges of species means that any process that affects species richness will also affect the species composition in grid cells and the large-scale congruence of species distributions. The processes behind species richness and species composition can thus not be decoupled at this scale, and importantly, employing classic regression methods that deal with spatial autocorrelation by imposing a symmetric neighborhood variance kernel (e.g., spatial autoregressive and conditional autoregressive) is not adequate to evaluate the effect of local processes on species richness. Other approaches, based on explicit mechanistic modeling of assembly processes, are likely to be necessary to resolve this.

The 1° × 1° represents the highest precision we can feasibly attain over such a large number of species but entails that many areas within each occupied grid cells will not actually be occupied by a given species and that species co-occurring at the grid cell scale may never actually co-occur in local communities. This is particularly pronounced along ecotone boundaries, where many species range boundaries are expected to meet, making it hard to evaluate patterns, e.g., along the Andes.

It has long been clear that analyses of species richness must move beyond simple correlative analyses of species richness numbers and take account of the size, shape, and location of the geographic ranges of species. By doing so here, we are better able to evaluate the postulated mechanisms and predictions of competing hypotheses on the origin of species richness patterns, providing a needed corollary to the template predictions of energy—kinetic theory. The ADF diagrams provide a visually intuitive way of turnover in species composition and the relationship among different assemblages. The empirical results of our study are consistent with recent developments in ecological theory that argue that dynamics at the scale of complete ranges must be considered in an integrative theory for species richness^32^. This entails a revision of species richness theory that expands geographical grid-cell-based regression analyses of diversity patterns to integrate species niches and geographic range dynamics.

## Methods

### Distribution data

The analyses were based on a distributional database of all species of birds, mammals, and amphibians in South America. The continent was divided into 1° × 1° grid cells corresponding to integer degree lines of latitude and longitude. All continental grid cells that contained land area inside were retained in the analysis, yielding a total of 1689 1° × 1° grid cells. Land-bridge islands (e.g., Trinidad) were excluded.

The bird distributions were extracted from the Copenhagen global bird database^33^, which is periodically updated. This database is based on museum specimens, published sight records, and expert opinion, following the approach outlined by Rahbek and Graves^5,34^. The distributions were mapped directly to the 1° × 1° latitude–longitude grid; hence, distributions are not based on a post hoc fitting of arbitrary-scale polygons to the grid cells (different from the mammal and amphibian datasets, which are based on IUCN data). The resulting high-quality dataset represents the best current knowledge of bird distributions in South America. To ensure comparability with other sources, the avian species delimitation followed the taxonomy published by the SACC^35^ resulting in a dataset containing 2869 land and freshwater species. For a full list of the >1600 references used to build the global dataset from which these distributions are derived, see the appendix of Holt et al.^22^

The mammal database was based on published range maps from the global mammal assessment^36^, as modified by Fritz and Purvis^37^. Domesticated species were excluded from the analysis, as well as portions of the geographic ranges of non-domesticated species labeled as historical, presence uncertain, introduced origin, or extinct. Species classified as Data Deficient, Extinct, or Extinct in the Wild in the IUCN database were excluded. The resulting database was updated to match the published phylogeny of mammals^38^. This entailed excluding 130 species that were absent from the phylogeny and adding 40 species from PanTHERIA^39^ that were missing from the IUCN database. Finally, 56 species were added by splitting IUCN maps to reflect the phylogeny of Bininda-Edmonds et al.^40^. Range polygons for the resulting 1146 mammalian species were overlaid on the 1° × 1° grid. A species was scored as present in any grid cell that overlapped the geographic range polygon for that species^41^.

The amphibian database was based on published range maps from the IUCN^42^ and updated to follow the published taxonomy of amphibians^43^. Species labeled as incertae sedis, klepton, and undescribed taxa were excluded, as were species classified as introduced or uncertain/introduced. The final dataset consisted of range maps for 2265 amphibian species, which were overlaid on the 1° × 1° grid and scored as present or absent in each grid cell. We note that the amphibian dataset is not quite of the quality of the other two datasets and have been revealed to fit poorly with occupancy records^44^. We have chosen to include it here for consistency across the tetrapod groups, but conclusions based on the amphibian dataset should be interpreted carefully.

### Environmental data

Records of mean annual temperature and annual precipitation were extracted from the mean monthly climatic database published by New and co-workers^45^, which was compiled globally at a 0.5° latitude–longitude resolution for the period 1961–1990 (>3,000,000 data points for each variable).

### Assemblage dispersion fields

The occurrence of species can be summarized in a presence–absence matrix **P**, where each row of the matrix represents a species, each column is a grid cell, and a 0 or 1 denotes absence or presence of a species within a grid cell^46^. The row sums represent the range sizes of species (measured as the total of occupied grid cells), and the column totals represent species richness values of grid cells. The ADF matrix **D** is then defined as

1 $${\mathbf{D}} = {\mathbf{P}}^{\mathrm{T}}{\mathbf{P}},$$

where T is the transpose operator. **D** is a symmetrical site-by-site matrix that counts the number of species shared by any two grid cells. Each row (or column) represents the ADF for that cell and describes the number of species that a focal cell shares with any other cell. Mapping dispersion fields results in the maps presented in Fig. 2 and Supplementary Figs. 2 and 3, representing the geographical pattern of biotic similarity around the selected focal cells.

The row (and column) sums of the ADF matrix equal the ADF volume of cells. The ADF volume measures the sum of shared species between a specified cell and all other cells, or, equivalently, the sum of the range sizes of all species present within the focal cell^26^. Thus, if *p*_*i,j*_ refers to the *i*th row and *j*th column of **P**, i.e., the presence of species *i* in cell *j*, and *r*_*i*_ is the range size of species *i*, defined as

2 $$r_i = \mathop {\sum}\limits_{j = 1}^N {p_{i,j}} ,$$

then the dispersion field volume is

3 $${\mathrm{df}}\_{\mathrm{volume}}_j = \mathop {\sum}\limits_{i = 1}^S {p_{i,j}r_i} ,$$

where *S* is the total number of species and *N* is the total number of grid cells. It also quantifies the mean contribution of a cell to all ADFs, since cells with a higher ADF volume share more species with surrounding cells. Dividing the dispersion field volume with the species richness of sites yields the mean range of all species present at the site, what Smith^47^ labeled the mean average cosmopolitanism of species.

### Measurement of ADF asymmetry

The center of gravit*y* of the dispersion field for a grid cell *j* was calculated as the point located at the average longitude (*x*) and latitude (*y*) of the ranges of all species present within cell *j*.

So the mean *x* and *y* coordinates of species *i* is

4 $$\overline {x_i} = \frac{1}{{r_i}}\mathop {\sum }\limits_{j = 1}^N p_{i,j}x_{i,j}$$

5 $$\overline {y_i} = \frac{1}{{r_i}}\mathop {\sum }\limits_{j = 1}^N p_{i,j}y_{i,j}$$

and then

6 $${\mathrm{df}}\_{\mathrm{centerX}}_j = \frac{1}{{s_j}}\mathop {\sum }\limits_{i = 1}^S p_{i,j}\overline {x_i}$$

7 $${\mathrm{df}}\_{\mathrm{centerY}}_j = \frac{1}{{s_j}}\mathop {\sum }\limits_{i = 1}^S p_{i,j}\overline {y_i}$$

Although the contours and symmetry of ADFs exhibit marked geographic variation, the total extents of ADFs (i.e., areas that share at least one species with the focal cell) are similar because most assemblages of birds, mammals, and amphibians contain a few widespread species that occur over most of the South American continent. For example, the Neotropic cormorant (*Phalacrocorax brasilianus*) occurs in every cell (*n* = 1689) in South America, so that all focal cells shared this species with all other grid cells. The pervasive influence of widespread species forces the center of gravity of ADFs toward the geographic center of South America and obscures regional signals of biotic similarity. This is particularly true in mammals (mean range size = 182.7 grid cells) and birds (mean range size = 186.0 grid cells) but less so in amphibians (mean range size = 37.5 grid cells).

To visualize the distinct pattern in ADFs, we trimmed away grid cells that shared only a few species with the focal cell. The cutoff value for trimming represents a balance between the ability to visualize core ADF asymmetries and the need to illustrate the distributional pattern of the entire species assemblage for a focal cell. Higher cutoff values reflect increasingly restrictive patterns of biotic similarity. We arbitrarily set the value at 0.5 for amphibians and increased the value to 0.6 for birds and 0.7 for mammals, which have larger average range sizes. We then calculated the center of mass and the ADF asymmetry vector for the trimmed ADF for each focal cell. The resulting spatial patterns of ADFs and asymmetry diagrams were qualitatively similar for all three vertebrate classes (as seen in Supplementary Figs. 4–6).

### Null and predictive models

We created null models by randomly placing continuous ranges on the South American continent, using a simple spreading dye algorithm^30^. This algorithm starts by selecting a random starting cell anywhere on the continent. The algorithm then selects a random cell adjacent to that occupied and adds it to the range. This process continues until the target range size is reached. The algorithm is implemented in R.

To create the null model ADFs, we built a null distribution of random ranges for each grid cells. Ranges were selected from all possible random ranges in South America that intersect the focal cell, maintaining the empirical range–size frequency distribution of that grid cell. This was achieved by first building a sampling population of random ranges for each possible range size between 2 and 1689 grid cells over the entire domain (the continent), so that all grid cells were intersected by at least 100 different random ranges of each range size. For each grid cell, we then picked random ranges from this sampling population according to the empirical range–size distribution of that grid cell and constructed the ADF.

The resulting ADFs clearly demonstrated an effect of the geometry of the South American continent, which was most pronounced for the lowest regions of ADFs (Supplementary Fig. 7a, b). When focusing on the areas with more shared species (yellow to red in the figures), the dispersion fields were highly symmetrical, with biotic similarity declining as a basic function of distance in all directions from the focal cell. The ADF symmetry diagram exhibited a simple pattern of all vectors pointing toward the center of the continent (Supplementary Fig. 7c). These patterns represent the baseline for comparing the results of ecological process.

To quantify the deviance of empirical ADFs from the null model, we calculated standardized effect sizes by repeating the sampling procedure 1000 times and calculating ADF symmetry values for all grid cells. The deviation between the empirical and simulated symmetry values was then calculated as (Eq. 7) (*ν* − *μ* (**λ**))/*σ* (**λ**), where *ν* is the empirical symmetry value, **λ** is a (mathematical) vector of symmetry values, and *μ* and *σ* are the mean and standard deviation of the simulated symmetry values, respectively.

We also implemented three different predictive models for the expected ADF symmetry patterns expected under various constraints on range origination and expansion. In model 1, constrained origination, we placed the first grid cell of ranges on the continent according to a probability function of available energy and allowed range expansion into each surrounding grid cell to be random. To generate model 3, constrained range expansion, we placed the first grid cell on a random grid cell in a vegetation ecoregion chosen with a probability proportional to the occupancy of that ecoregion by a given species. Random range expansion was allowed, but a range expansion event into a grid cell across a boundary was 30× less likely than an expansion event into a grid cell in the same ecoregion (the number 30 was chosen to ensure that species’ ranges expanded across boundaries occasionally, but rarely). Sampling from these models followed the protocol outlined above. Model 2, constrained species richness, was generated in a different way, which was designed to maintain the grid cell richness constant, reflecting a scenario where the richness of each grid cell is restricted to a maximum value by local resources. All species ranges were built simultaneously, at each step allowing one species to expand its range by one grid cell adjacent to its range, with a probability equal to the difference between the maximum (i.e., the empirical) species richness of the grid cell and the current richness at that point in the simulation. This allows building random ranges while both maintaining range cohesion and the empirical species richness. At the end of the simulation, absolute range cohesion would become impossible, and small gaps were allowed (as exists in the empirical ranges as well). ADF diagrams were built for all three null models (Fig. 5).

### Ecotone analysis for Amazonia

It is a prediction of niche conservatism that the asymmetry of ADF vectors for cells within a major vegetation ecoregion should decrease with distance from the ecotone boundary. Because the scale of the distribution data (1° × 1° latitude/longitude degrees) was too coarse to adequately assess this effect for many of the smaller ecoregion, e.g., along the Andes, we tested the conjecture for the largest coherent region, Amazonia. To avoid the confounding influence of disjunct faunas that come into contact in grid cells at ecotones, we restricted the analysis to only include cells that are completely within the lowland (<500 m above sea level) Amazonian ecoregion and also excluded a small number of cells that were isolated behind the highland Tepuis of the Guianan Shield. The cells that were included in the analysis, along with their distance to the outer boundary of Amazonia, are shown in Supplementary Fig. 8. Residuals from the regression analysis were symmetrical but departed significantly from normality according to Kolmogorov–Smirnov test (*p* < 0.01).

### Reporting summary

Further information on research design is available in the Nature Research Reporting Summary linked to this article.

## Supplementary information

Supplementary Information

Reporting Summary

## Data Availability

All data depicted in figures here are available on request from the authors. The underlying databases with range maps on amphibians and mammals are available from the IUCN. The result of computer models are also available upon request.

## Code availability

All R code necessary to produce the results here will be made available upon acceptance. In addition, R and Julia codes necessary to use the ADF general computational framework are available as part of the nodiv package for R (on CRAN) (functions *dfield_matrix*, *dfield_explore*, *dfield_calc*, and *arrowplot*) and the SpatialEcology.jl package for Julia (can be installed from the official repository, function dispersionfield), both openly available on GitHub on the MIT free and open license.
