# Supplementary material for: Dispersion fields reveal the compositional structure of South American vertebrate assemblages
Source: Nat Commun. 2020 Jan 24;11:491. doi: 10.1038/s41467-019-14267-y (PMC6981175; doi:10.1038/s41467-019-14267-y)
Supplement: Supplementary file 1 — Supplementary Information [file 41467_2019_14267_MOESM1_ESM.pdf]

“Assemblage dispersion fields reveal the spatial structure of biotic similarity among South American vertebrate distributions”

Borregaard, M.K., Graves, G. & Rahbek, C.

Submission to Nature Communications 2019

Supplementary Figures

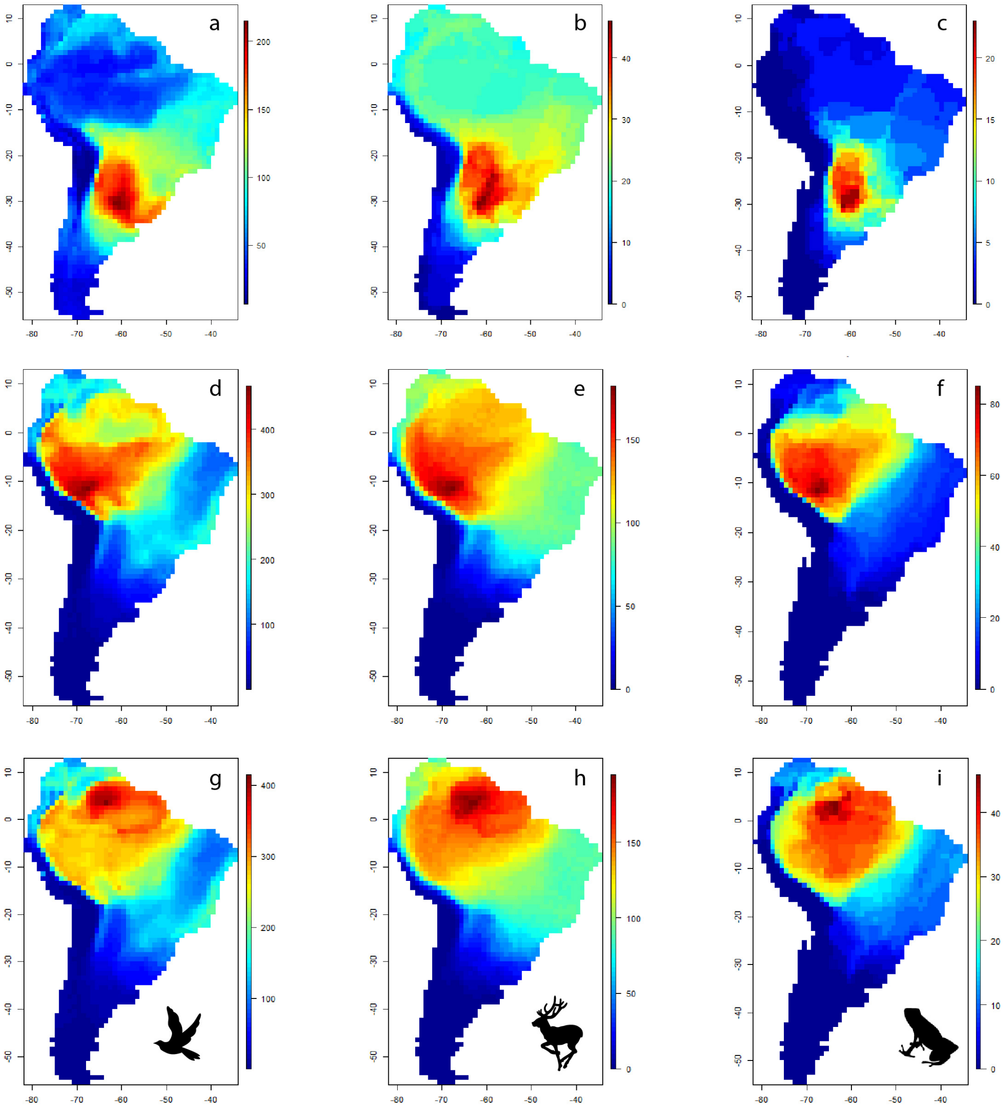

Supplementary Figure 1. Examples of dispersion fields from different grid cells. Each row shows the dispersion field for a given grid cell for birds, mammals and amphibians. The centroids of the focal cells are: row 1 (a-c): 61.5W 30.5S; row 2 (d-f): 63.5W 3.5N; row 3 (g-i): 68.5W11.5S.

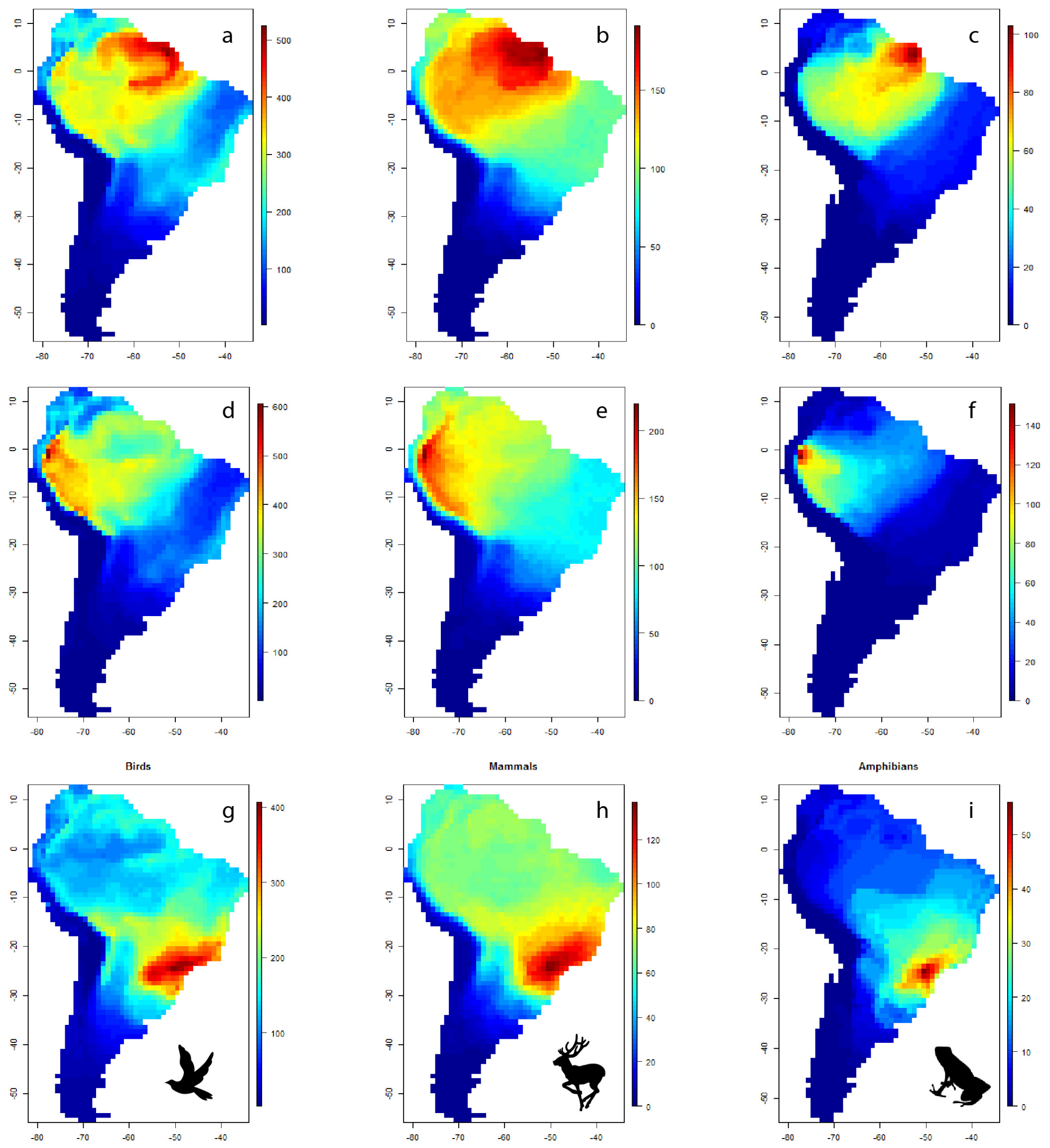

Supplementary Figure 2. Further examples of dispersion fields from different grid cells. Each row shows the dispersion field for a given grid cell for birds, mammals and amphibians. The centroids of the focal cells are: row 1 (a-c): 52.5W 3.5N; row 2 (d-f): 7 7.5W 1.5S; row 3 (g-i) 50.5W 24.5S.

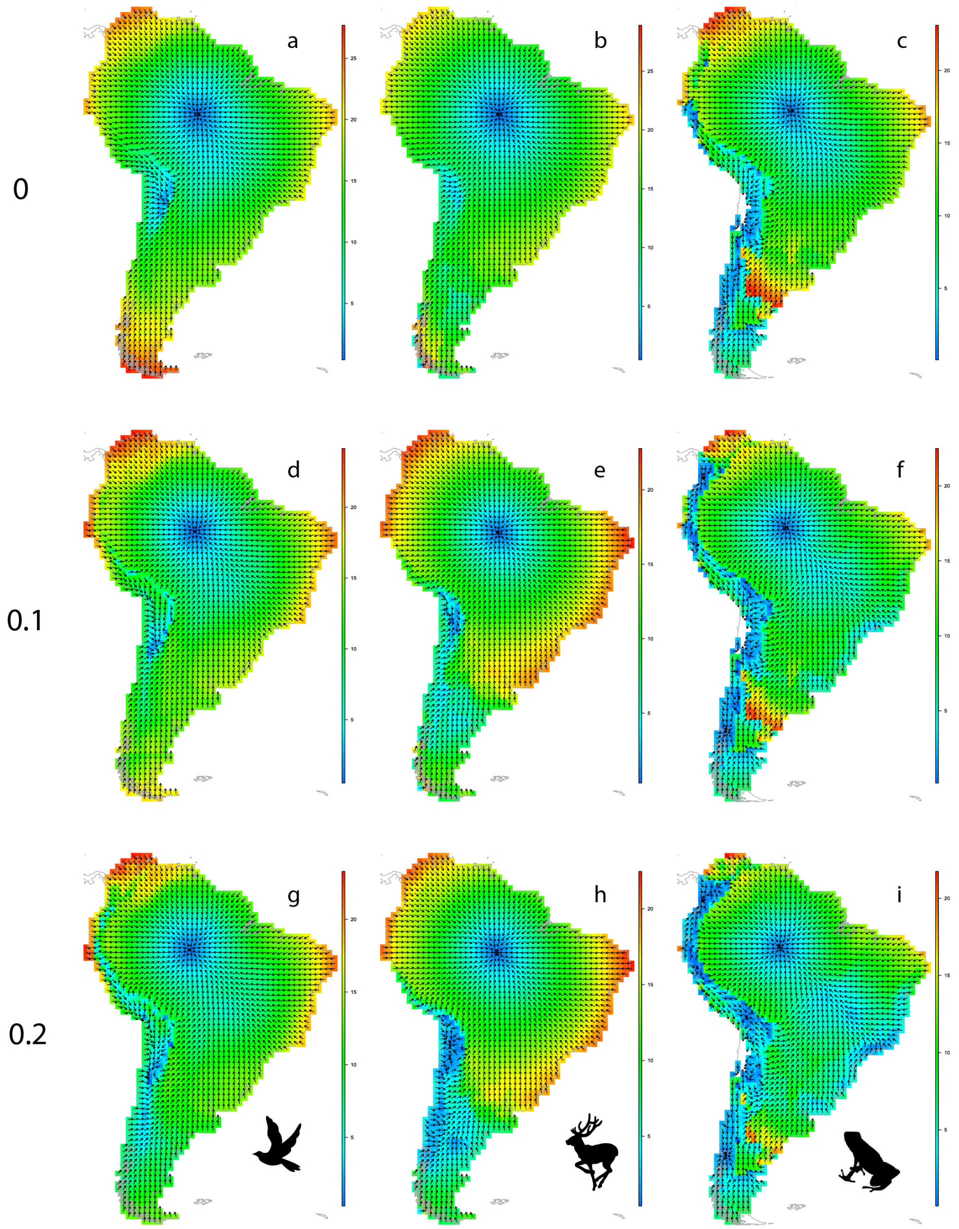

Supplementary Figure 3. An illustration of the dependence of *ADF* symmetry diagrams on the cutoff value used to delimit the central region of the *ADF*. The number at the left of each row indicates the cutoff value. A value of 0 indicates that all grid cells are used in calculating the centre of gravity of the *ADF*, whereas a value of 0.1 indicates that all grid cells that contain at least 10% of the species present in the focal cell are considered part of the central region. Each row shows the *ADF* symmetry diagrams for birds (a, d, g), mammals (b, e, h) and amphibians (c, f, i).

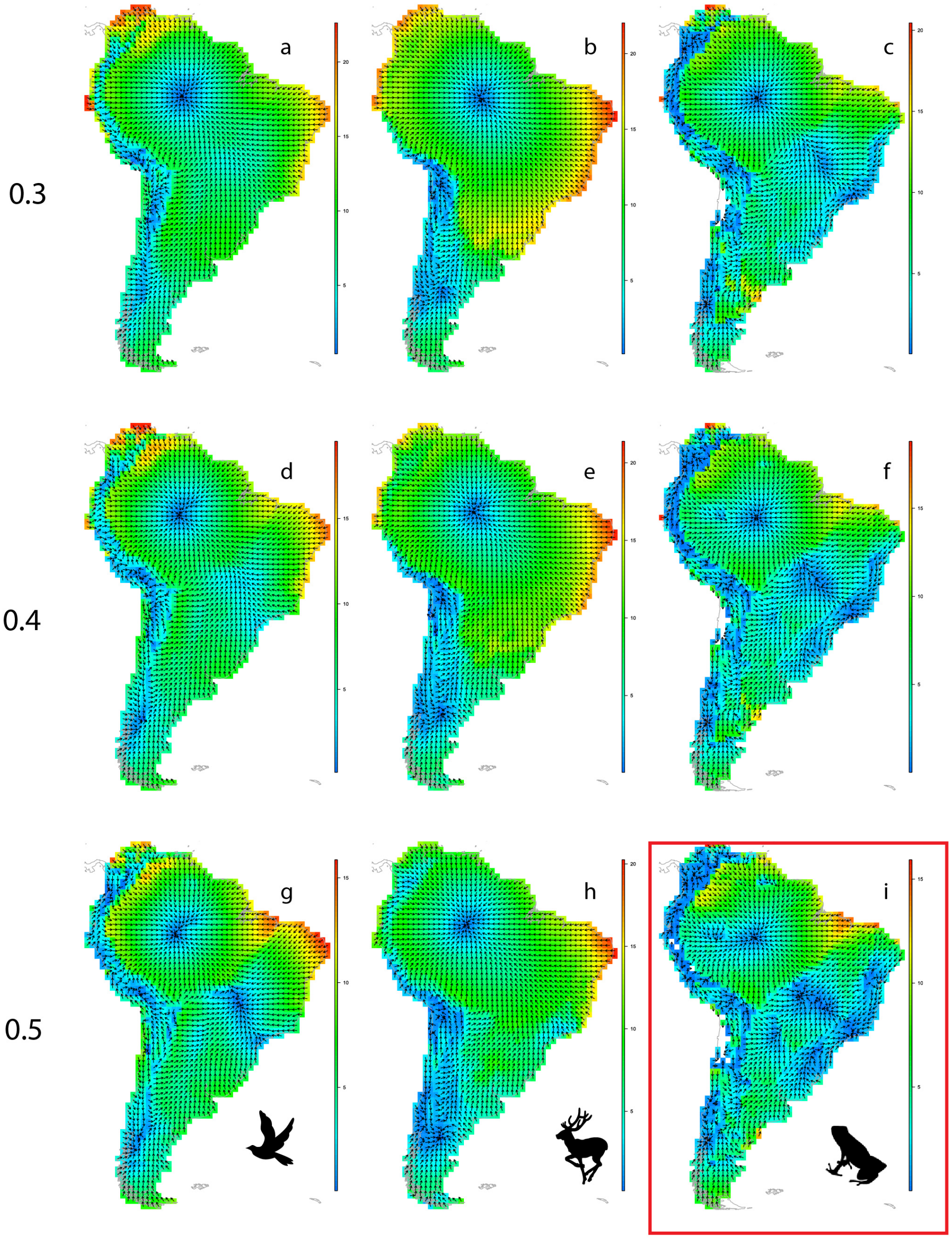

Supplementary Figure 4. ***ADF*** **symmetry diagrams for higher cutoff values.** The red box indicates the diagram that was included in the main text for this taxon. Legend as in Supplementary Fig 3.

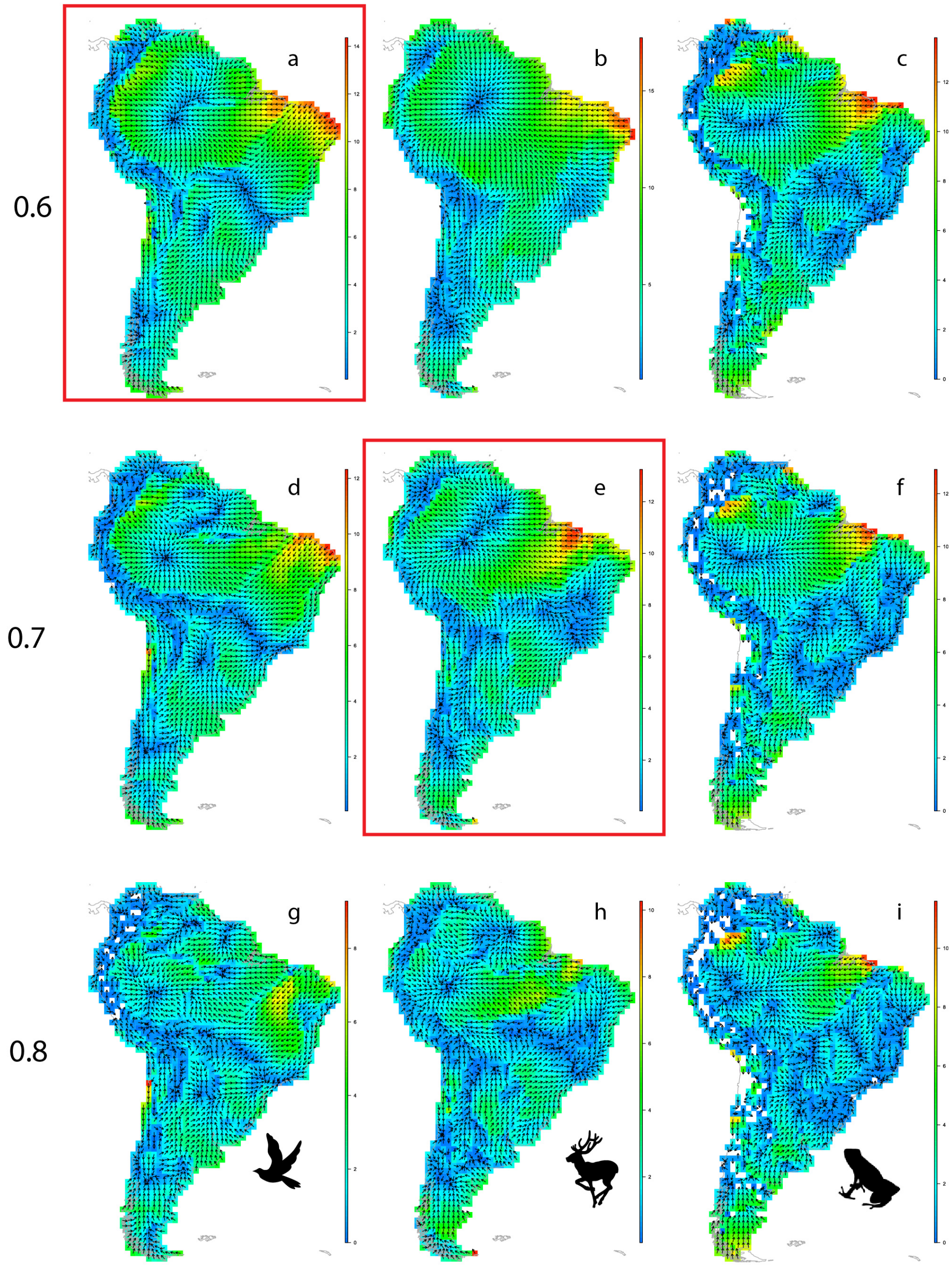

Supplementary Figure 5. ***ADF*** **symmetry diagrams for the highest cutoff values.** Legend as in Supplementary Fig 3.

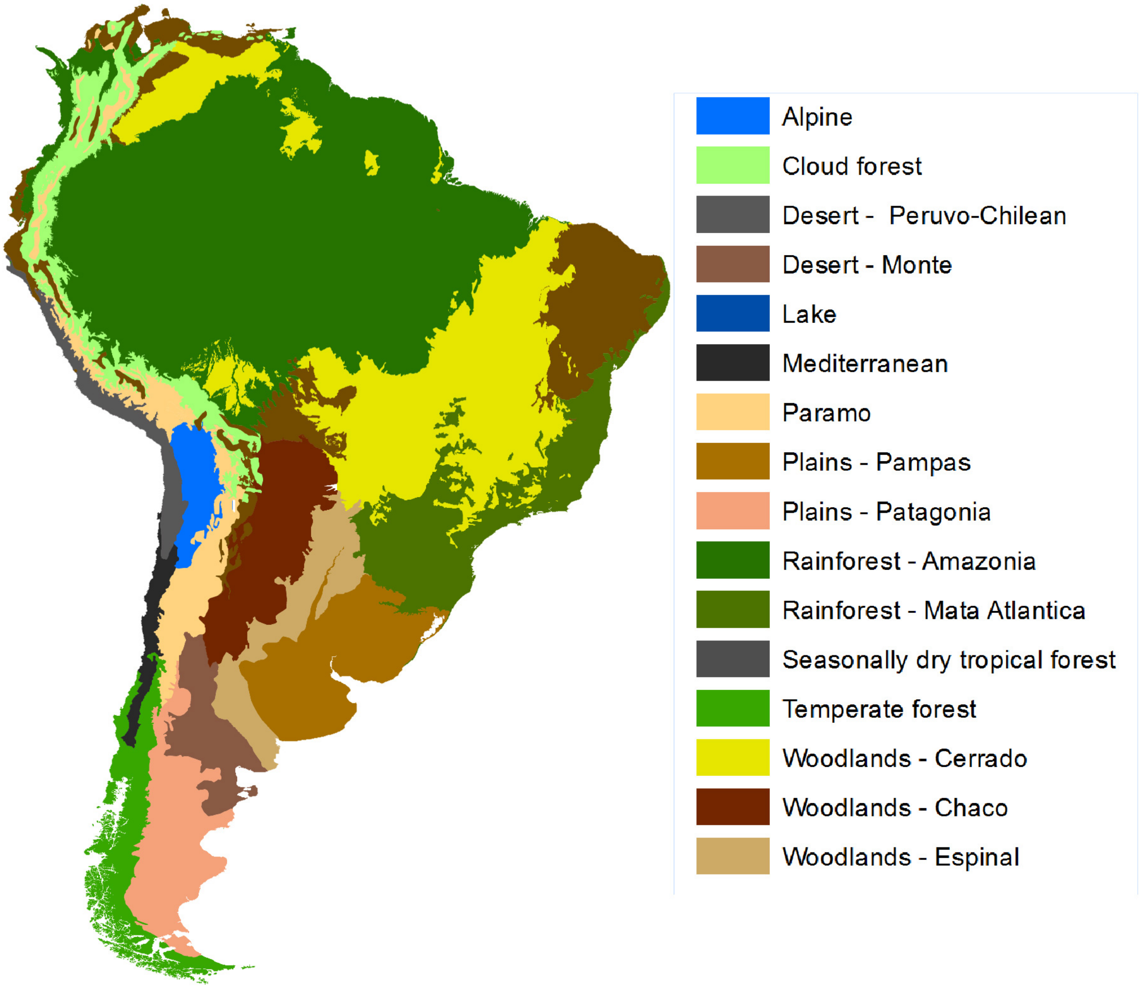

Supplementary Figure 6. Larger map of South American biomes. The map was provided by Tiina Särkinen, originally based on the map of the World's ecoregions developed by the WWF (under CC BY 4.0 license: https://creativecommons.org/licenses/by/4.0/).

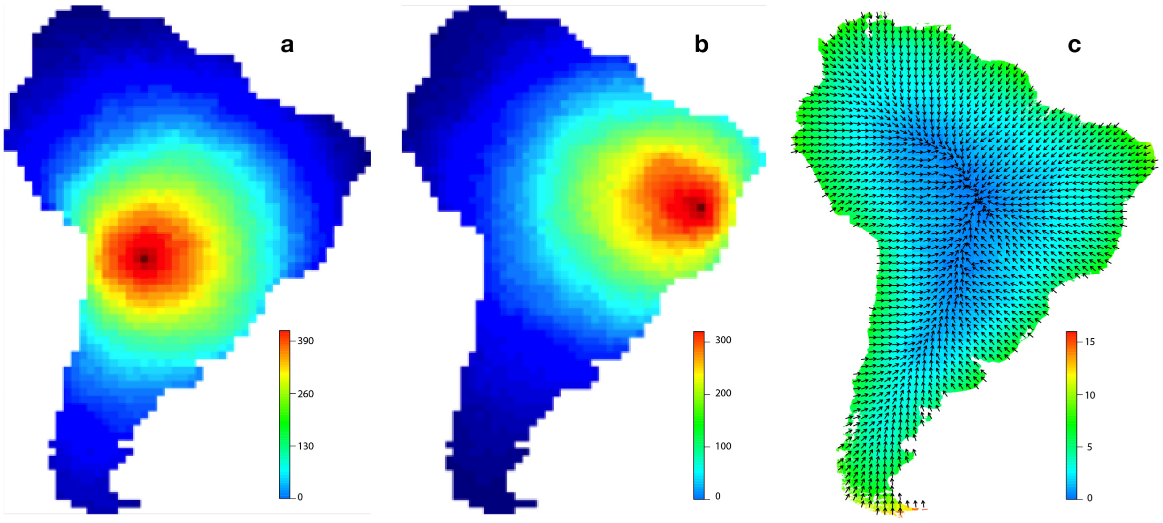

Supplementary Figure 7. Results from a basic spreading dye null model.
(a-b) Examples of dispersion fields created by the spreading dye model. Biotic similarity is an approximate function of distance, similarly to the predictions of the species-energy model. (c) The *ADF* symmetry diagram expected under a null model of cohesive, non-interacting ranges. All symmetry vectors are oriented towards the centre of the continent.

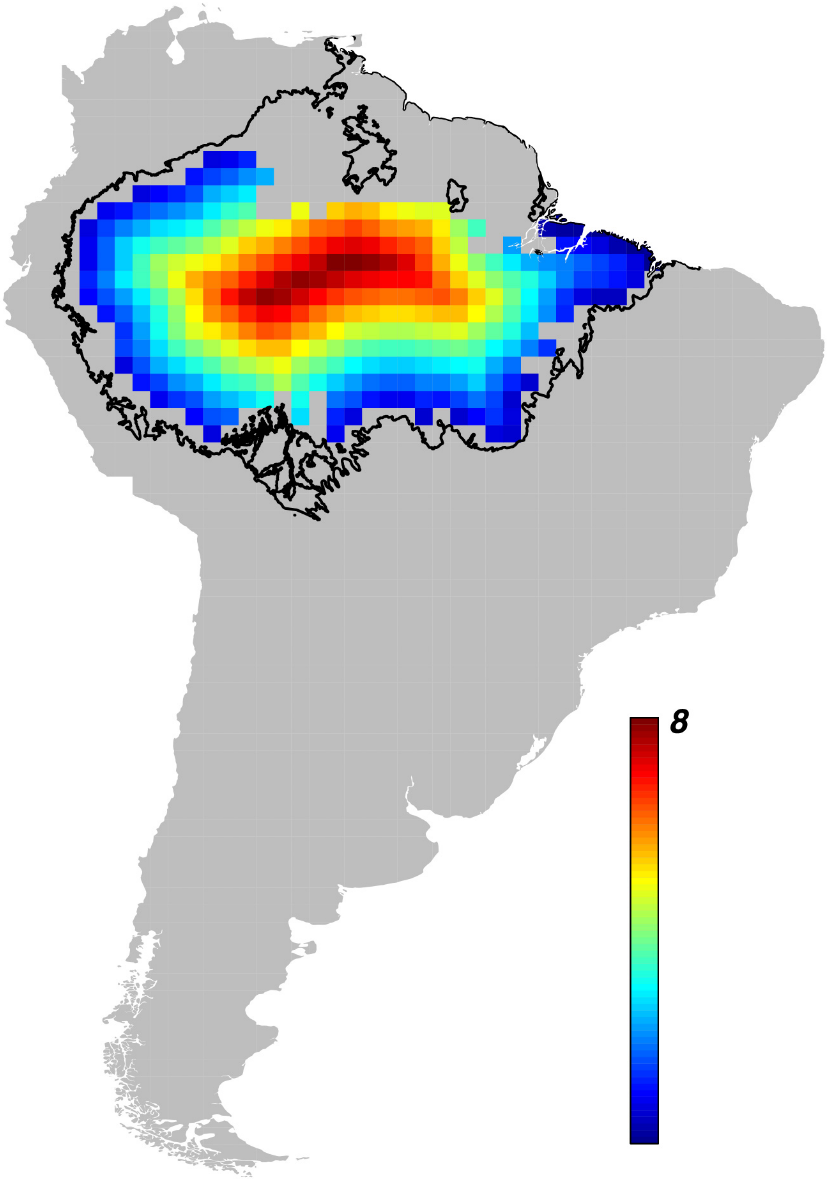

Supplementary Figure 8. The cells used for the analysis of the correlation between ecotone distance and *ADF* symmetry. The ecotone around Amazonia is shown as a black line. All grid cells intersecting the ecotone are excluded, as well as all cells with a maximum altitude over 500meters, which marks a high-altitude fauna that is distinct from the Amazonian.
